# Telomere Length in Preterm Infants: A Promising Biomarker of Early Adversity and Care in the Neonatal Intensive Care Unit?

**DOI:** 10.3389/fendo.2017.00295

**Published:** 2017-10-31

**Authors:** Livio Provenzi, Giunia Scotto di Minico, Roberto Giorda, Rosario Montirosso

**Affiliations:** ^1^0–3 Center for the At-Risk Infant, Scientific Institute IRCCS Eugenio Medea, Lecco, Italy; ^2^Molecular Biology Laboratory, Scientific Institute, IRCCS Eugenio Medea, Lecco, Italy

**Keywords:** adversity, epigenetics, neonatal intensive care unit, pain, preterm birth, stress, telomere

## Abstract

Preterm infants present an immature neurobehavioral profile at birth, even in absence of severe brain injuries and perinatal complications. As such, they require a long-lasting hospitalization in the Neonatal Intensive Care Unit (NICU), which is thought to grant at-risk newborns’ survival, but still entails a number of physical, painful, and socio-emotional stressors. Hence, preterm birth and NICU stay represent an early adverse experience, which has been linked to detrimental consequences for neurological, neuro-endocrinal, behavioral, and socio-emotional development, as well as to disease later in life. Recent advances in the behavioral epigenetic field are helping us to unveil the potential mechanisms through which early NICU-related stress may lead to negative developmental outcomes. From this perspective, telomere regulation might be a key programming mechanism. Telomeres are the terminal portion of chromosomes and are known to get shorter with age. Moreover, telomere length (TL) is affected by the exposure to stress during early development. As such, TL might be an innovative biomarker of early adverse exposures in young infants and children. Unfortunately, there is paucity of studies investigating TL in populations of preterm infants and its association with known NICU-related stressors remains unexplored. In the present paper, the potential relevance of TL for research and clinical work with preterm infants will be underlined in the light of recent contributions linking progressive telomere shortening and early exposure to adverse experiences and stressful environments in humans. Finally, insights will be provided to guide clinically relevant translational research on TL in the field of VPT birth and NICU stay.

## Introduction

Preterm birth (<37 weeks of gestational age) is estimated to account for about 11% of all live births worldwide (1). During the last decades, biomedical progress has greatly improved the survival rates of prematurely born infants. Notwithstanding, preterm birth still constitutes a major health problem worldwide (2). Notably, despite preterm infants’ morbidity is highly affected by the extent of brain damage and immaturity and other related clinical conditions, preterm infants’ health and disease is at least partially affected by the stressful nature of the precocious hospitalization in the Neonatal Intensive Care Unit (NICU) (3).

Telomeres are repeat sequences at the ends of chromosome arms, known to shorten because of cellular aging and in relation to disease conditions (4). Recent studies highlighted that stress greatly influences telomere erosion by shaping the biochemical structure in ways that may promote telomere damage, inflammation, and greater rate of leukocyte division, in part through the impairment of telomerase-mediated elongation, but also through other pathways (5, 6). Unfortunately, the effects of early stress exposure on telomere length (TL) in hospitalized preterm infants remain unexplored. Here, we discuss how TL might be an emergent research field with potential innovative insights for researchers and clinicians involved in the neonatal care of preterm infants.

## Preterm Birth and Nicu Stay as an Early Adverse Experience

Preterm infants need long-term hospitalization and specialized multi-disciplinary interventions in NICU. During this time, they are exposed to painful procedures (7, 8) and high-intensity physical stimulation (e.g., lights and sounds exposure) which represent sources of stress affecting the development of the brain and of the neuroendocrine system (9, 10). As a result, preterm birth is associated with heightened risk of long-term detrimental consequences for development, including sensorial, behavioral, neurological, and emotional disorders (11, 12). Even in absence of critical comorbidities, NICU procedures are distressful for premature newborns, leading to long-lasting programming of less-than-optimal developmental trajectories (13–15).

### Sources of NICU-Related Stress

The NICU environment is far from being a surrogate of the maternal womb and represents a source of distress in newborns due to three main aspects: (1) sensorial adverse stimulation, (2) invasive and painful procedures, and (3) limited opportunities for parental caregiving and alteration of parent–infant bonding.

First, infants are exposed to physical and sensorial stimulations (i.e., lights and sounds) characterized by levels of intensity and length of exposure which easily exceed the average recommended exposure standard for newborns (10). As such, NICU sensory conditions contribute to physiological instability in preterm infants, causing long-term harmful consequences for visual and auditory development later in life (16).

Additionally, invasive and painful clinical procedures (e.g., intubations, venipunctures, arterial insertions, and surgery) are needed to improve the health status of preterm infants. Due to their immature neurobehavioral profile, preterm infants present lower threshold and higher sensitization to external perturbations, so that even routine handling (e.g., diaper change) might be responded to with a heightened physiological response (17). Interventions such as skin-breaking procedures have been associated with detrimental consequences for short- and long-term development (18, 19), encompassing structural and functional alterations of brain development, which persist throughout childhood (20). Furthermore, the level of NICU-related pain exposure is associated with neurodevelopment, including motor and cognitive development (21) as well as emotional problems during later childhood (22). These developmental alterations are partially mediated by an altered functioning of the main system of stress response, namely the hypothalamic–pituitary–adrenal (HPA) axis. Several studies documented that cumulative exposure to skin-breaking pain during NICU stay might associate with altered resting cortisol levels (15), increased stress reactivity (13), and altered HPA regulation (23) in prematurely born subjects, independently from clinical confounders.

Notably, preterm newborns are suddenly separated from the mother after birth, which may disrupt the biological routes to caregiving and parental bonding (24) and alter HPA axis reactivity to pain stimulations (25).

### NICU Stay and Developmental Care (DC)

During the last decades, DC strategies have been developed to minimize the negative effects of preterm birth, optimize infants’ neurobehavioral development, and improve interactions between parents and infants (26, 27). DC includes the management and reduction of external stimuli (e.g., vestibular, auditory, visual, tactile), the facilitation of parental presence, and involvement in early care of the infant (e.g., kangaroo care, a technique in which infants are placed in skin-to-skin contact with a parent, usually on the breast), the facilitation of breastfeeding, and pain management (e.g., non-pharmacological analgesia such as non-nutritive sucking, glucose, containment with towels or blankets) (28). These interventions are meant to promote parent-to-infant bonding and to promote better outcomes in terms of infants’ neurobehavioral stability (e.g., nesting, swaddling, prone positioning).

The quality of DC is critical to counterbalance the effect of NICU-related stressors on preterm infants’ developmental trajectories. Preterm infants hospitalized in NICUs characterized by high quality of DC practices exhibit better neurobehavioral development during the first month of life (29), less internalizing behaviors at 18 months (30), and improved language outcomes at 36 months (31). Specific DC interventions, such as kangaroo care, are associated with improved physiologic stability (32), sleep organization (33), brain maturation (34), and behavioral–emotional development (35).

## Preterm Behavioral Epigenetics: In Search of Biological Mechanisms

Despite NICU-related stress has well-known impact on preterm infants’ development, little is known about the underlying biological mechanisms. Recent scientific advances suggest that epigenetic mechanisms are highly sensitive to environmental stimulations and might play a pivotal role in the embedding of early NICU-related adversities into the developing trajectories of preterm infants. DNA methylation of stress-related genes [e.g., *NR3C1*, encoding for cortisol receptors and *SLC6A4*, encoding for the serotonin transporter (36)] is affected by NICU-related pain exposure (37, 38). Moreover, these epigenetic markers have been linked with long-lasting developmental trajectories in behavioral and socio-emotional domains during infancy (39, 40) and childhood (37).

Notwithstanding, epigenetic mechanisms include a wide set of biochemical DNA modifications other than DNA methylation and telomere shortening represents another potential mechanism that has received limited attention in the field of prematurity. Here, we provide a perspective on the possibility that the NICU-related stress might exert stress-dependent telomere erosion.

### TL as a Cellular Aging Biomarker

**Figure 1:**
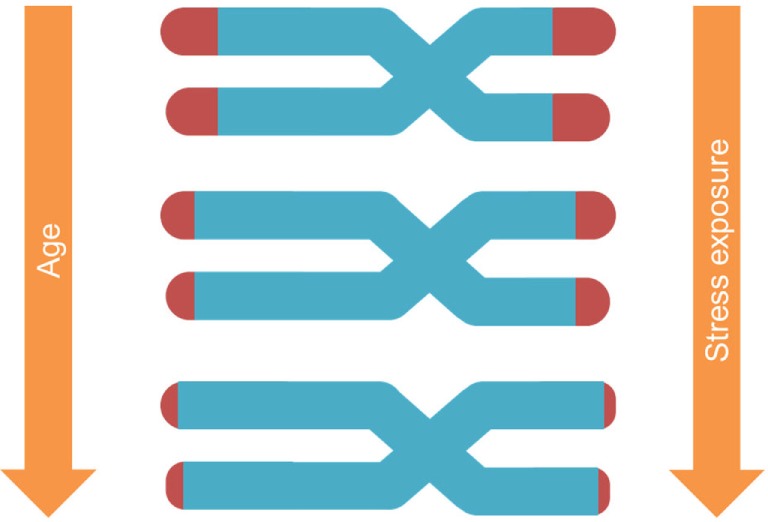
Telomere shortening is affected both by age and stress exposure.

Telomeres are the DNA–protein structures capping each chromosome end, regulated by the enzyme telomerase (41). With each cell replication, telomeres shorten until they reach a certain limit, after which the cell enters a state of arrest (Figure 1). Notably, TL depends on (a) its initial setting at birth and (b) the magnitude of telomere erosion from birth onward (42). Telomere erosion, in turn, depends on cell replication rate, cumulative exposure to agents that produce DNA damage (such as oxidative, inflammatory, endocrine, and other forms of biological stress), and activity of the telomerase enzyme (43). Shortened TL and/or reduced telomerase activity have been associated with health risk and diseases (44–46).

### TL as an Early Adversity Exposure

Telomere length regulation and telomerase activity are influenced by environmental and behavioral factors (6). Animal studies documented that manipulation of maternal diet during pregnancy has an effect on offspring TL among different tissues and organs (47). In chickens, prenatal administration of corticosteroids in the yolk resulted in greater telomere erosion and increased duration of stress response in the offspring compared to a control group (48).

Human studies also documented associations between telomere regulation and high levels of psychosocial stress exposure (49–51), suggesting that stress-related changes in telomere integrity may be one possible mechanism linking psychosocial stress and age-related disease (52). Entringer and colleagues (53) recently reported an association between maternal exposure to severe psychosocial stress during pregnancy and TL of children during young adulthood. Moreover, pregnancy-related maternal stress predicted shorter newborn leukocyte TL (5). Additionally, many studies found that exposure to adverse conditions in early postnatal life such as severe social deprivation (54), maltreatment (55, 56), and PTSD (57) is associated with subsequent shortening of telomeres.

## Implications of TL for Preterm Infants Stress and DC

In light of the evidence reported above, analysis of TL can be a technique that might reveal scientifically intriguing and clinically relevant insights into early developmental risk detection and to better predict developmental outcomes of preterm infants. Based on the literature reviewed, one might hypothesize that preterm infants might present a pronounced risk of early telomere erosion. Specifically, in the next sections, we provide an overview of (1) current evidence pertaining to TL analysis and prematurity; (2) methodological issues; and (3) implications for research and clinical practice.

### TL Analysis and Prematurity: Current Evidence

To best of our knowledge, only few studies have investigated TL in the preterm infants. One study reported no significant differences between mean leukocyte TLs of preterm infants compared with their full-term counterparts at birth (58). However, very low birth weight preterm infants (<1,500 g) had significantly longer telomeres than low birth weight ones (1,500–2,500 g), and gestational age appeared to be a significant mediator of telomere restriction. Moreover, this study was cross-sectional, 15 preterm and 11 full-term newborns were included and no longitudinal evidence of telomere erosion was provided. In a subsequent prospective study, five preterm infants and eight age-matched fetuses were followed longitudinally for 8–12 weeks and consistent telomere erosion was observed in preterm infants between 23 and 35 weeks of gestation (59). In a larger sample study, Menon and colleagues (60) compared TLs of infants following either spontaneous preterm birth with intact membrane, or preterm birth due to premature rupture of membrane (pPROM) and full-term infants. In intact-membrane pregnancies, fetal leukocyte TL was inversely proportional to gestational age. Moreover, newborns from intact-membrane gestations had longer telomeres compared to both full-term infants and preterm infants following pPROM.

In a more recent longitudinal study, TL was assessed at birth in both preterm and full-term infants and at term-equivalent age in preterms only (61). Preterm infants were found to have longer telomeres compared to full-term counterparts. Nonetheless, consistent with previous research (58, 60), TL erosion increased with advancing gestational age at birth in a limited subgroup of infants (*N* = 5). Vasu and colleagues (61) concluded that the longer telomeres measured in the preterm infants’ sample at birth might be due to a period of high cell turnover and replicative stress which occurs during the final weeks of pregnancy for full-term infants, but not for prematurely born ones. Despite current results are non-conclusive and further research is needed, this evidence suggests that altered TL regulation might play a role in preterm infants’ status.

### Methodological Issues

Previous TL research in preterm infants mainly focused on birth and/or term-equivalent age. Moreover, as reported by Vasu and colleagues (61), the samples of previous works are relatively small so that the studies might be underpowered with limited capacity to detect true associations between prematurity-related conditions and telomere erosion. In addition, to date, no study has inquired the effects of NICU-related stress and DC practices on telomere regulation in preterm infants. In the following sections, we review specific issues implied by research on TL in preterm infants and provide insights for future research.

A major issue in measuring TL is the availability of many methods to assess telomere erosion (62) such as TRF analysis, quantitative RT-PCT, single telomere length analysis (STELA), and quantitative FISH (Q-FISH). To the best of our knowledge, there is no rationale for choosing one as a gold standard as each method presents different strengths and limitations. They vary in terms of training needed and equipment costs (low in TRF and high in Q-FISH and quantitative RT-PC), risk of artifacts and variability (e.g., slot blot analysis has limited risk of artifacts, but provides highly variable data, particularly with small samples), and capacity to provide TL information on global DNA (e.g., quantitative RT-PCR) or single chromosomes (e.g., STELA). As such, the choice of method should take into consideration all these aspects as well as the aims of the research study. Notably, the presence of studies using diverse techniques require relevant efforts to replicate these findings in future research (62).

Another critical point is that preterm infants constitute a heterogeneous population, varying on perinatal (e.g., birth weight, gestational age, clinical complications, etc.), socio-demographic (e.g., socio-economic status), and contextual factors (e.g., NICU quality of DC). This implies that researchers need to pay attention to different conditions that interact in a complex manner in affecting TL in preterm infants. For instance, preterm infants born at lower gestational age might have longer telomeres at birth but greater stress-related erosion due to cumulative NICU-related stress and pain. Consistently, careful selections of preterm infants in terms of inclusion criteria should be pursued to avoid mixed samples of subjects, which might affect the quality of life, developmental trajectories, and number of stressful intervention to which they are exposed during the NICU stay.

Differences in DC practices among diverse NICUs and countries represent another source of variation (63). Despite the lack of information on epigenetic effects associated with DC (64), different levels of care are known to be related to different developmental outcomes (65). Future research should take into account the existence of differences in DC quality when investigating telomere regulation in preterm infants, possibly using a multi-centric study design involving different NICUs.

Finally, the context and timing of early adversity exposure might be critical in setting a TL-related biomarker of later-in-life health and disease (5, 66). As such, longitudinal and prospective research designs should be considered the golden standard when looking at epigenetic variations and TL. The model of preterm newborns and infants constitutes a naturally occurring condition in which stress exposure is expected and thus can be measured and monitored at different time points through different epigenetic biomarkers (67).

### Implications for Research and Clinical Practice

**Figure 2:**
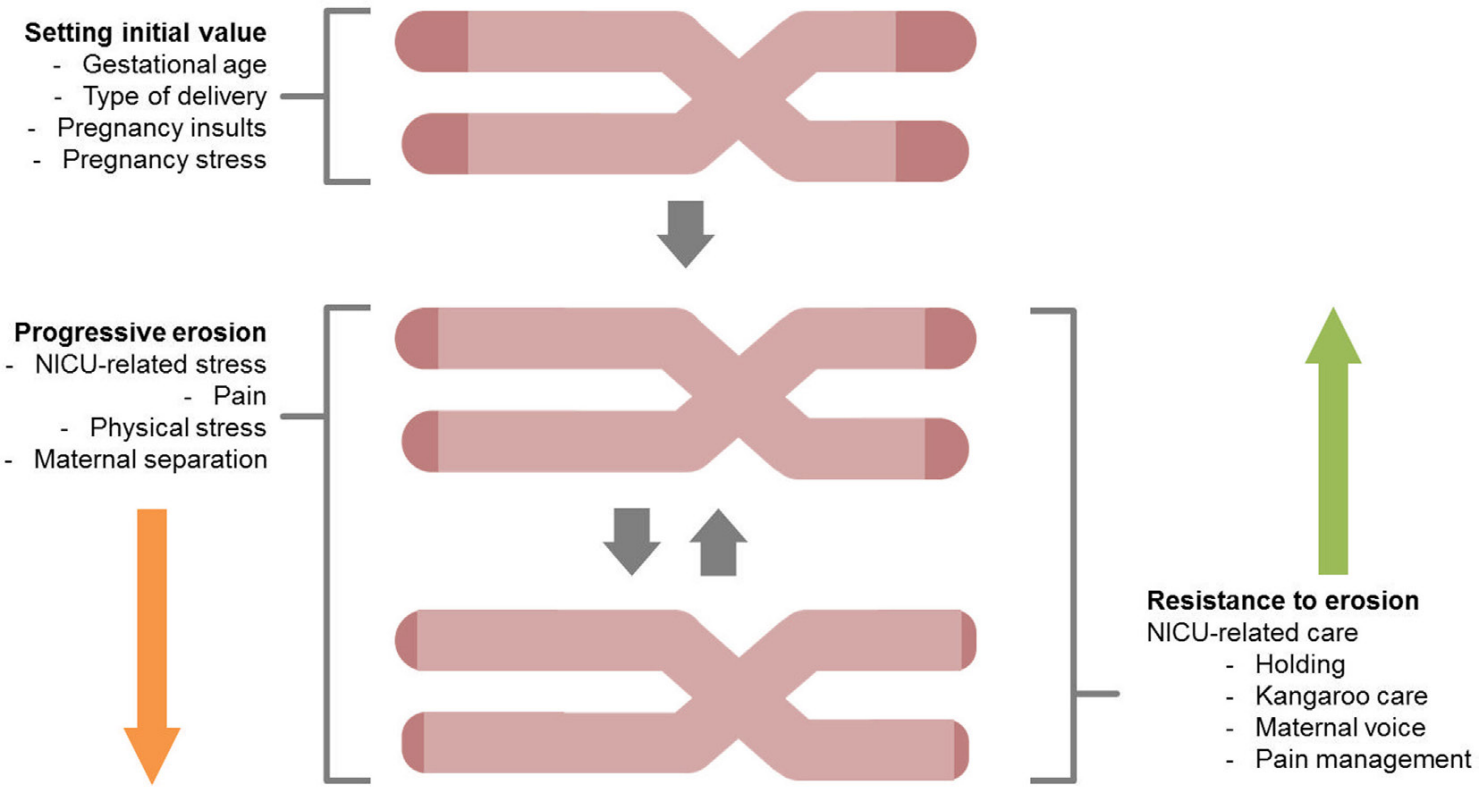
A schematic overview for the study of telomere length shortening in preterm infants. Both stressful effects (progressive erosion) and protective factors (resistance to erosion) are highlighted.

**Figure 3:**
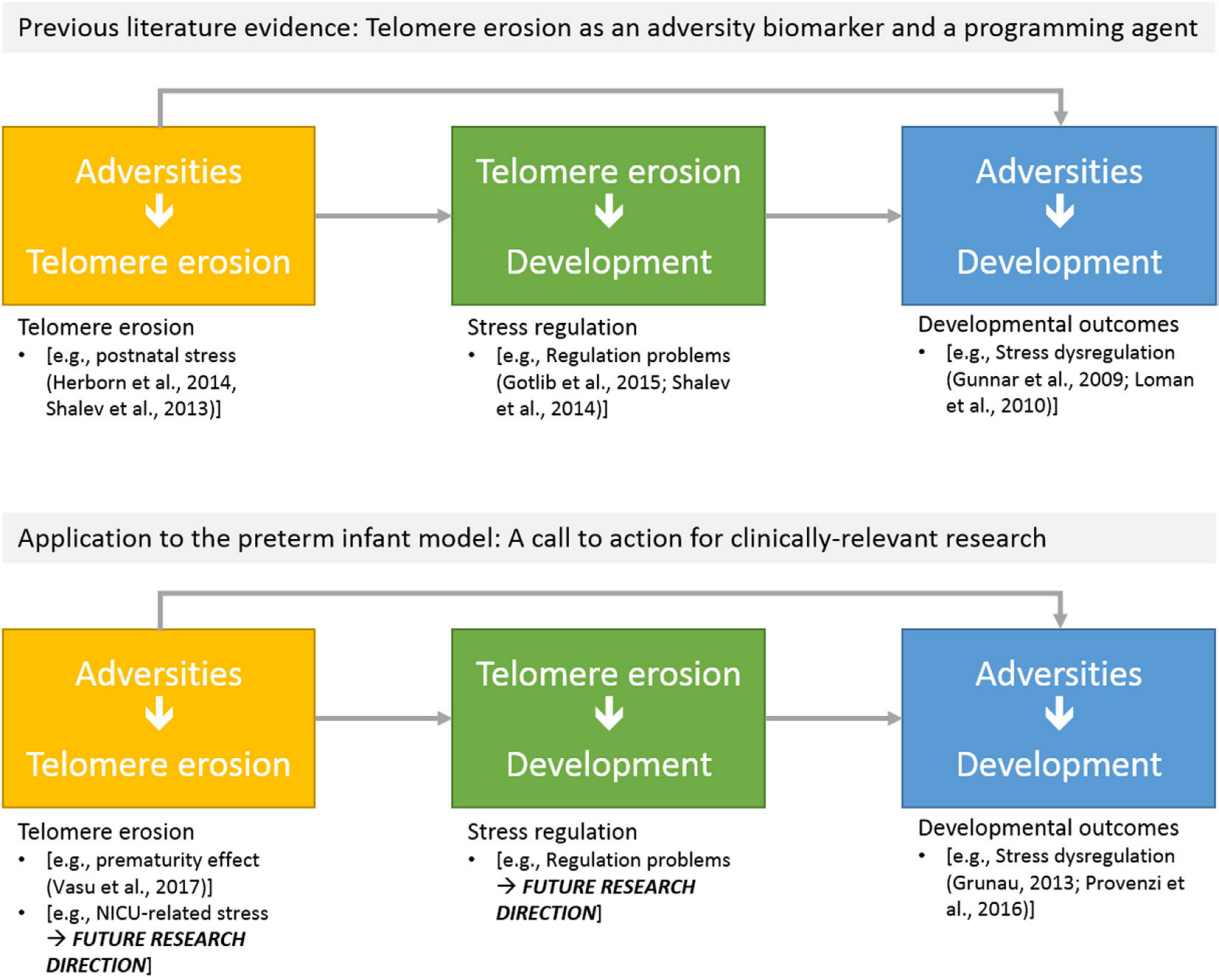
Schematic overview of potential research directions on telomere length erosion in preterm infants: (a) effects of NICU-related adversities on telomere erosion; (b) the predictive role of telomere erosion on developmental outcomes.

Figure 2 reports major areas of future investigations in this field. First, as previous research on animal models (68) and human adults (66) documented that stress is highly involved in increased erosion of telomeres, one might wonder whether NICU-related stress has an impact on preterm infants’ TL from birth to discharge. Notably, a key component of NICU-related stress is represented by invasive and painful stimulations [e.g., skin-breaking procedures and invasive mechanical ventilation (7)]. Cumulative pain exposure in NICU has epigenetic effects on stress-related genes (38, 69). A direct link between pain and telomere regulation in humans has been found in patients with osteoarthritis: those with high levels of pain had shorter telomeres compared to the counterparts reporting low pain (70). Moreover, preterm infants are at risk for HPA axis dysregulation (23). Notably, children exposed to early adverse experiences (i.e., maternal depression) not only exhibit shorter telomeres compared to controls but increased TL erosion was associated with heightened HPA axis reactivity to a stress-inducing laboratory procedure (71). Consistently, direct investigation of the association among NICU levels of pain exposure (e.g., number of skin-breaking procedures), rate of TL shortening and the HPA axis dysregulation represent an interesting path for future research (see Figure 3).

Future investigation could also consider the protective effects of DC practices in reducing or slowing the rate of telomere erosion. Studies in adult human subjects have demonstrated that positive changes in life-style have the possibility to result in significantly increased telomerase activity and improved telomere maintenance capacity in human immune-system cells (72). Similarly, one could hypothesize that positive changes in the NICU, in term of healing environment (e.g., reduction of sensorial adverse stimulation), infant pain management, increased presence of parents, facilitation of early interaction and parent–infant bonding, might result in a relenting of telomere erosion in preterm infants.

## Conclusion

The study of TL in preterm infants is an intriguing area of research and a potential source of scientifically sound beneficial information for NICU staff. Although now it remains a hypothetical scenario, it is possible to speculate that TL measure might have some clinical implications for NICU-related stress and infant DC. For example, given the evidence for association between TL and pain (70), one could expect that TL might be used as a marker of the effect of pain exposure in preterm infants. It is also intriguing to hypothesize that TL could be used to document the biological correlates (by either decreasing the erosion rate or improving the turnover of telomerase activity) of the beneficial effects of DC practices, such as kangaroo care and infant/caregiver closeness. From this perspective, the investigation of the role of early environment in affecting TL regulation dynamics is warranted to be a pivotal area of research in future preterm infants’ research, especially within the emergent area of preterm behavioral epigenetics field of study (36). In sum, despite the field of preterm behavioral epigenetics is still at its beginning (73), TL could become a biomarker of developmental risk in both high- and low-risk preterm infants, holding promises of providing NICU staff with innovative index to predict which infants are at increased risk of less-than-optimal developmental outcomes.

## Author Contributions

LP conceived the work, wrote the first draft of the manuscript, and approved the final version of the paper. GSM contributed to literature review, revised English language, and approved the final version of the paper. RG supervised the work, by providing unique expertise in relation to biological sections, and he approved the final version of the paper. RM contributed to the conception of the work, supervised the writing, and approved the final version of the paper. All authors agree to be accountable for all aspects of the work.

## Conflict of Interest Statement

The authors declare that the research was conducted in the absence of any commercial or financial relationships that could be construed as a potential conflict of interest.
